# *Nelumbo nucifera* Seed–Derived Nitrogen-Doped Hierarchically Porous Carbons as Electrode Materials for High-Performance Supercapacitors

**DOI:** 10.3390/nano11123175

**Published:** 2021-11-23

**Authors:** Lok Kumar Shrestha, Rekha Goswami Shrestha, Rashma Chaudhary, Raja Ram Pradhananga, Birendra Man Tamrakar, Timila Shrestha, Subrata Maji, Ram Lal Shrestha, Katsuhiko Ariga

**Affiliations:** 1 International Center for Materials Nanoarchitectonics (WPI-MANA), National Institute for Materials Science (NIMS), 1-1 Namiki, Tsukuba 305-0044, Ibaraki, Japan; MAJI.Subrata@nims.go.jp (S.M.); ARIGA.Katsuhiko@nims.go.jp (K.A.); 2 Amrit Campus, Tribhuvan University, Kathmandu 44613, Nepal; chaudharyreshma896@gmail.com (R.C.); rajaram2620@gmail.com (R.R.P.); timilastha@gmail.com (T.S.); 3 Tri-Chandra Multiple Campus, Tribhuvan University, Kathmandu 44600, Nepal; tamrakar_birendra@hotmail.com; 4 Department of Advanced Materials Science, Graduate School of Frontier Sciences, The University of Tokyo, 5-1-5 Kashiwanoha, Kashiwa 277-8561, Chiba, Japan

**Keywords:** *Nelumbo nucifera* seed, KOH activation, hierarchically porous carbon, nitrogen-doping, energy storage, supercapacitor

## Abstract

Biomass-derived activated carbon materials with hierarchically nanoporous structures containing nitrogen functionalities show excellent electrochemical performances and are explored extensively in energy storage and conversion applications. Here, we report the electrochemical supercapacitance performances of the nitrogen-doped activated carbon materials with an ultrahigh surface area prepared by the potassium hydroxide (KOH) activation of the *Nelumbo nucifera* (Lotus) seed in an aqueous electrolyte solution (1 M sulfuric acid: H_2_SO_4_) in a three-electrode cell. The specific surface areas and pore volumes of Lotus-seed–derived carbon materials carbonized at a different temperatures, from 600 to 1000 °C, are found in the range of 1059.6 to 2489.6 m^2^ g^−1^ and 0.819 to 2.384 cm^3^ g^−1^, respectively. The carbons are amorphous materials with a partial graphitic structure with a maximum of 3.28 atom% nitrogen content and possess hierarchically micro- and mesoporous structures. The supercapacitor electrode prepared from the best sample showed excellent electrical double-layer capacitor performance, and the electrode achieved a high specific capacitance of ca. 379.2 F g^−1^ at 1 A g^−1^ current density. Additionally, the electrode shows a high rate performance, sustaining 65.9% capacitance retention at a high current density of 50 A g^−1^, followed by an extraordinary long cycle life without any capacitance loss after 10,000 subsequent charging/discharging cycles. The electrochemical results demonstrate that *Nelumbo nucifera* seed–derived hierarchically porous carbon with nitrogen functionality would have a significant probability as an electrical double-layer capacitor electrode material for the high-performance supercapacitor applications.

## 1. Introduction

Supercapacitors, or electrical double-layer capacitors (EDLCs), store charges in the form of electrical double-layers at the electrode surface by the diffusion of electrolyte ions from the electrolyte solution, are the current state-of-the-art energy-storage systems for the storage of electrochemical energy [1,2,3,4,5,6,7,8,9,10]. Recently, supercapacitors have attracted significant attention because of their enormous high power density (>400 kW kg^−1^), extremely rapid charging or rapid reversible adsorption/desorption of electrolyte ions at the electrode surface, extraordinary long cycling stability without any capacitance loss (>10,000), high rate performance, low-cost and easy operation, as well as environmentally friendliness [11,12,13,14,15,16,17]. However, despite the several merits supercapacitors suffer from, the low energy density (1–20 Wh kg^−1^) compared to the lithium-ion batteries (>160 Wh kg^−1^), limiting their practical applications in devices.

The energy density is proportional to the electrode material’s specific capacitance (*C*_s_) and the potential operating window of the cell (*V*). To improve the energy density, one has to either increase the *C*_s_ of the electrode material or widen the potential window. The working potential window or voltage can be achieved by replacing electrolyte solution with aqueous to non-aqueous solvents. Moreover, ionic liquids can also be used, as they can be operated over a more comprehensive voltage range than water [18,19,20,21]. However, due to the safety issue, non-aqueous solvents or ionic liquids are less explored in practical supercapacitor devices, demonstrating the need to improve the electrode material’s textural properties. Electrode materials for supercapacitors require a large specific surface area and plenty of well-defined pores for the adsorption of large numbers of electrolyte ions so that the energy storage capacity could be enhanced significantly. Therefore, nanoporous materials with a subtle balance of micro- and mesopore structure and high conductivity have become promising electrode materials in supercapacitors applications. Microporosity contributes to enhancing the electrical double-layer formation, while mesoporosity promotes the electrolyte ions’ diffusion and contributes to enhancing the rate performance. Investigations have shown that pore size distribution is a critical factor in controlling the effective surface area. The materials with hierarchically porous structures consisting of both the micro and mesopore architectures display better performance as electrode materials than the other conventional materials [22,23,24,25,26].

Among the various nanoporous materials studied, activated carbon materials due to high specific surface areas and abundant pores, high electrical conductivity and heteroatom doping display the high specific capacitance [27]. Such micro- and mesoporous carbon with nitrogen functionality can accommodate more electrolyte ions and increase the performance of the electrode materials in supercapacitor applications. Several nanoporous carbon materials such as fullerenes or fullerene crystals-derived porous carbons, carbon fibers, carbon nanotubes (CNTs), reduced graphene oxides and metal–organic framework (MOF)-derived porous carbon materials have been explored as the electrode materials of supercapacitors [28,29,30,31,32,33,34,35,36,37,38]. These carbon materials show good capacitive behavior, but the limitation in the scale-up synthesis sustainability of the energy storage system is low.

Nanoporous activated carbons prepared from biomass or biopolymers are the leading supercapacitor electrode materials due to the ultrahigh surface area, large pore volumes, good electrical conductivity, high chemical and thermal stability, simple preparation method and low cost [39]. Besides, heteroatom doping in the biomass-derived carbon further improves the performance of the energy-storage applications [40]. The major component of the biomass is the lignocellulose, which upon pyrolysis at moderate temperature (~200 to 300 °C), transforms to porous biochar. The biochar can be further activated by mixing with chemical activating agents such as KOH, zinc chloride, and phosphoric acid and carbonized at higher temperatures (up to 1000 °C) to enhance the porosity and the specific surface area. Several biomass-derived nanoporous carbons have been reported to show high surface area above 2000 m^2^ g^−1^, depending on the precursor itself, carbonization temperature, chemical activating agent and mixing ratio with the activating agent [41,42,43,44,45,46]. Due to high surface area and well-developed porosity, the biomass carbons perform excellently as the electrode materials for the electrochemical supercapacitors. For example, *Choerospondias axillaris* (Lapsi) seed–derived carbon material exhibited a high specific surface area of ca. 2272.3 m^2^ g^−1^ with interconnected mesoporous structure, and the electrode showed excellent supercapacitance performance giving a high specific capacitance of ca. 284 F g^−1^ at a current density of 1 A g^−1^ with a high rate performance sustaining 67.7% capacitance at 20 A g^−1^ and long cycle stability retaining 99% capacitance after 10,000 charging/discharging cycles [47]. Activated carbons are also equally explored in the hybrid capacitor to enhance electrochemical energy storage. For example, Minakshi and co-worker [48] reported a hybrid capacitor comprising mixed transition-metal sodium phosphate/activated carbon, which exhibited a specific discharge capacitance of 45 F g^−1^ over 1000 cycles. In a recent study, Zhu and co-workers [49] reported nitrogen-doped (5 at%) hierarchically porous carbon from biodecomposited products with an exceptionally high surface area of 3142 m^2^ g^−1^. Due to the improved textural properties, the material showed outstanding performance as the electrode materials for supercapacitor applications giving a high specific capacity of 209 F g^−1^ at 0.05 A g^−1^. However, a capacity loss of about 9% was observed after 10,000 cycles. Similarly, Wu and co-workers [50] successfully fabricated unique porous microrods from albizia flowers, and the derived material possessed hierarchically porous architectures. The materials showed a high surface area of 2757.6 m^2^ g^−1^, and self-nitrogen doping of 1.34 wt% was found. Furthermore, the albizia flowers-derived porous microrods showed a high specific capacitance of 406 F g^−1^ at 0.5 A g^−1^. In a separate work, Chen and co-workers [51] also fabricated the self-nitrogen-doped three-dimensional (3D) nanoporous carbon materials from waste cottonseed husk with honeycomb-like interconnected hierarchical porous structures. Due to a suitable surface area of 1694.1 m^2^ g^−1^, the cottonseed husk carbon material performs excellently as the electrode material for supercapacitors. The electrode showed a high specific capacitance of 238 F g^−1^ at 0.5 A g^−1^ with a relatively good cycle stability of 91% after 5000 charging/discharging cycles. Wickramaarachchi and co-workers [52] have recently reported KOH-activated carbon material from a bio-waste, Mango seed husk. The material obtained by the carbonization at 1100 °C showed the best textural properties with a high specific surface area of 1943 m^2^ g^−1^ and an average pore volume of 0.397 cm^3^ g^−1^. As a result, the carbon exhibited a maximum capacitance of 135 F g^−1^ at 5 mA cm^−2^ with the energy density of 19 Wh kg^−1^ at the power density of 1077 W kg^−1^. We recently found that Lotus seed yields nanoporous carbon material upon activation with zinc chloride with moderate surface areas and pore volumes (1103–1316 m^2^ g^−1^ and 0.741–0.887 cm^3^ g^−1^) [53]. Furthermore, the electrode prepared from the optimal sample performs reasonably well as the electrical double-layer capacitor achieving the specific capacitance of 272.9 F g^−1^ at 1 A g^−1^ indicating the possibility of further enhancing the energy storage capacity by optimizing the surface textural properties of Lotus-seed–derived carbon materials. These examples demonstrate the importance of biomass for producing electrode materials with good textural properties and self-nitrogen-doping for enhancing the overall properties of the supercapacitors.

In this work, we synthesized self-nitrogen-doped ultrahigh surface area nanoporous carbon material with hierarchically micro- and mesoporous structures from *Nelumbo nucifera*–seed powder and studied its electrochemical energy storage performance, the electrode material for the supercapacitor in an aqueous electrolyte solution (1 M H_2_SO_4_) in a three-electrode cell. The fabrication method includes mixing biochar of *Nelumbo nucifera* seed in potassium hydroxide (KOH) and carbonization at higher temperatures from 600 to 1000 °C in an inert atmosphere of nitrogen. Due to the high surface area, well-developed porosity, nitrogen doping and partially developed graphitic carbon structure, the electrode prepared from the optimal sample showed excellent electrical double-layer capacitor performance achieving a high specific capacitance of ca. 379.2 F g^−1^ at 1 A g^−1^ current density followed by a high rate performance of 65.9% at a high current density of 50 A g^−1^ and extraordinary long cycle life without any significant capacitance loss after 10,000 charging/discharging cycles. These results indicate the potential of *Nelumbo nucifera* seed as the natural precursors for the large-scale and cost-effective production of the hierarchically porous nitrogen-doped activated carbon materials essential for the development of sustainable electrode material for high-performance supercapacitors.

## 2. Materials and Methods

### 2.1. KOH Activation of Nelumbo Nucifera Precursor

After adequately washing with Milli-Q filtered water *Nelumbo nucifera* (Lotus) seed was dried at 100 °C for 24 h and crushed to powder form, using a mechanical crusher. The Lotus-seed powder (100 g) was heated at 300 °C for 6 h under the air atmosphere to obtain biochar. The yield of the biochar was ca. 44%. The biochar (1 g) was then mixed with KOH pellet at a 1:1 weight ratio and ground and stored at 25 °C for 24 h. The mixture was carbonized at high temperatures, from 600 to 1000 °C, under a nitrogen gas atmosphere. The temperature ramp, hold time and nitrogen gas flow were 5 °C min^–1^, 3 h and 120 cc min^−1^, respectively. The carbonization was performed in a tube furnace (KOYO, Tokyo, Japan). After the carbonization, the obtained samples were mixed with a dilute hydrochloric acid solution (0.5 M); stirred for 3 h, using the magnetic stirrer; and washed with pure water, several times, until the solution attained a neutral pH of 7. After drying in vacuum at 80 °C for 12 h, all the samples were ground into powders and designated as LTSC_K600, LTSC_K700, LTSC_K800, LTSC_K900 and LTSC_K1000, where the numbers indicate the carbonization temperature. After the carbonization, the mass of material was found to be 0.26 g (LTSC_K600), 0.24 mg (LTSC_K700), 0.23 g (LTSC_K800), 0.21 g (LTSC_K900) and 0.18 g (LTSC_K1000). For comparison, Lotus seed (1 g) was directly carbonized at 800 °C without KOH and designated as LTSC_800. After the direct carbonization, 0.24 g of the LTSC_800 was obtained. The KOH activation for the generation of pore structure in carbon materials is a well-known phenomenon. It includes the etching of the carbon skeleton to develop pores through gas production and washing off the potassium compounds. The KOH activation involves the following reactions (the reduction of K compounds, oxidation of C and other intermediate reactions):

6KOH + C → 2K + 2K_2_CO_3_ (1)

K_2_CO_3_ → K_2_O + CO (2)

CO_2_ + C → 2CO (3)

K_2_CO_3_ + 2C → 2K + 3CO (4)

K_2_O + C → 2K + CO (5)

### 2.2. Characterizations of Nelumbo nucifera–Derived Nanoporous Carbons

The prepared nanoporous activated carbon materials were subjected to advanced characterizations, including thermogravimetric analysis (TGA) (SII Instrument, Model Exstar 600, Tokyo, Japan), Fourier-transform infrared (FTIR) (Nicolet 4700, Thermo Electron Corporation, Waltham, MA, USA) spectroscopy, powder X-ray diffraction (XRD) (Rigaku X-ray diffractometer, RINT, Tokyo, Japan), Raman scattering (NRS-3100, JASCO, Tokyo, Japan), X-ray photoelectron spectroscopy (XPS) (Theta Probe spectrometer, Thermo Electron Co. Karlsruhe, Germany) and scanning electron microscopy (S-4800, Hitachi Co., Ltd. Tokyo, Japan). In addition, the textural properties, including specific surface area and pore-volume and pore-size distributions, were estimated by nitrogen sorption measurements (Quantachrome Autosorb-iQ2, Boynton Beach, FL, USA).

### 2.3. Electrochemical Studies

Using cyclic voltammetry (CV), galvanostatic charge/discharge (GCD) and electrochemical impedance spectroscopy (EIS), we studied the electrochemical performances of the Lotus-seed–derived nanoporous carbon materials as the electrode materials for supercapacitor applications. Working electrodes were prepared on the glassy carbon electrode (GCE: outer and inner diameters of 10 and 5 mm, respectively). First, carbon material was dispersed in a mixed solvent (water:ethanol = 4:1, 2 mg mL^−1^) and sonicated for 60 min. Next, the suspension (3 µL) aliquot was drop cast at the center of the GCE (5 mm diameter) and dried at 60 °C for 2 h for the evaporation of solvents, which gave the mass of the active material on the electrode is 6 × 10^−3^ mg. Finally, a Nafion solution (5% in ethanol: 5 µL) was added on top of the carbon sample on GCE as a binder and further dried under reduced pressure at 80 °C for 12 h. In addition, a platinum wire and Ag/AgCl were used as counter and reference electrodes, respectively. The EIS measurements were performed at an amplitude of 5 mV, in the frequency range of 0.01 Hz to 100 kHz. All the electrochemical measurements were carried out in a three-electrode system in an aqueous electrolyte of 1 M H_2_SO_4_ at 25 °C. CHI 660E workstation (CH Instruments, Inc. Austin, TX, USA) was used. The specific capacitance (*C*_s_) of the electrode material was calculated from both CV (Equation (6)) and GCD curves (Equation (7)), using the following equations.

(6) $$C_{s}=\frac{1}{m\times v\times \Delta V}\int_{V_{1}}^{V_{2}}I\left(V\right)dV$$

where *m*, *v*, *V* and *I* represent mass of the electrode material (g), scan rate (mV s^−1^), operating potential window (V) and current (A), respectively.

(7) $$C_{s}=\frac{I\times t_{d}}{m\times \Delta V}$$

where *I*, *t*_d_, *m* and Δ*V* respectively represent the discharge current (A), the discharge time (s), the mass of the electrode material (g) and the potential window (V), respectively.

## 3. Results

**Figure 1:**
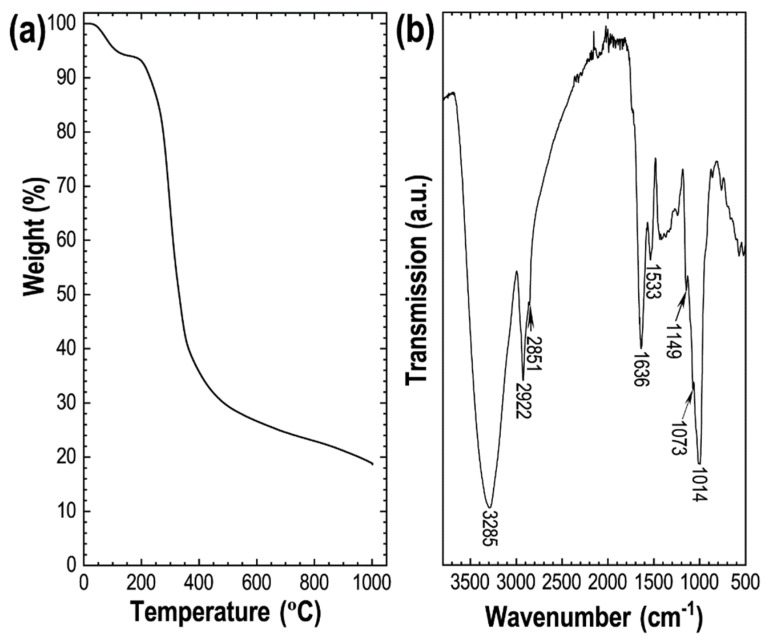
(**a**) TGA curve and (**b**) FTIR spectrum of the carbon source, Lotus-seed powder.

The pyrolysis properties of the carbon source, Lotus-seed powder (Figure 1a: TGA curve), reveal the carbonization process in different stages. The first stage involves the evaporation of moisture or crystallized water in the precursor below 200 °C. The polymerization of the main components of biomass, cellulose and hemicellulose takes place in the second stage, which also involves the degradation of carbohydrates and lipids in the range of 200–500 °C, releasing the volatile gases and thus causing a significant weight loss. As a result, about 70% of the mass is lost (Figure 1a). Finally, carbon formation occurs above 500 °C, with no noticeable weight loss in the TGA curve, demonstrating that the carbonization can be carried out above this temperature. Based on the pyrolysis characteristics, we conducted KOH activation of Lotus-seed biochar by carbonizing at different temperatures, namely 600, 700, 800, 900 and 1000 °C, where the carbon yields are estimated at 26.6, 24.5, 22.9, 21.2 and 18.8%, respectively.

**Figure 2:**
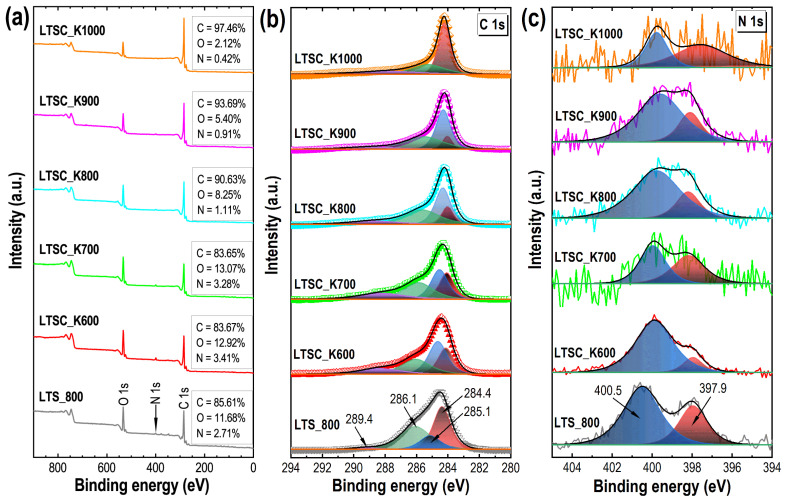
XPS surface composition studies of the Lotus-seed–derived carbon materials. (**a**) XPS survey spectra, (**b**) XPS C 1s spectra with the deconvoluted peaks and (**c**) XPS N 1s spectra with deconvoluted peaks of the directly carbonized sample LTS_800 and KOH-activated LTSC_K600, LTSC_K700, LTSC_K800, LTSC_K900 and LTSC_K1000 samples.

The FTIR spectrum shows the presence of heteroatoms (oxygen and nitrogen) functionalities in the precursor (Figure 1b). An intense FTIR peak at 3285 cm^−1^ corresponds to the N–H (str.), bands at 2922 and 2851 cm^−1^ corresponding to cellulose’s aliphatic C–H (str.). The band at 1636 cm^−1^ relates the O–H (def.) of adsorbed moisture water in the precursor. The FITR bands in the range of 1600–100 cm^−1^, commonly observed in the biomass due to cellulose and lignin, correspond to C–H (def.) and C–O (str.). After carbonization, the intensity of the FTIR bands corresponding to oxygen and nitrogen functionalities decreases significantly due to high-temperature carbonization (Supplementary Materials Figure S1). The broadband at 3446 cm^−1^ corresponds to adsorbed water, while a weak peak at about 1630 cm^−1^ can be attributed to the C=C (str.) common in the biomass-derived activated carbons [54]. The surface composition of the directly carbonized sample and the KOH-activated carbon materials was further investigated by the XPS (Figure 2). The XPS survey spectra display peaks at 284, 400 and 532 eV, which confirm carbon, oxygen and nitrogen as the main components of the prepared carbon materials (Figure 2a). Note that an increase in carbonization temperature alters the surface composition changes, and less oxygen and nitrogen functionalities are present in the samples carbonized at higher temperatures. Maximum nitrogen content of 3.4 at% was observed in LTSC_K600. The C 1s spectra of the samples could be deconvoluted into four peaks, with a peak centered at 284.4, 285.1, 286.1 and 289.4 eV, which correspond to C=C, C–N, C–C and C=O bonding states of the carbon material, respectively. The deconvoluted N 1s peaks centered at 397.9 and 400.5 eV, respectively, correspond to pyridinic-N and graphitic-N [55]. The O 1s XPS spectra with the deconvoluted peaks are shown in Supplementary Materials Figure S1b the two primary bonding states of oxygen.

**Figure 3:**
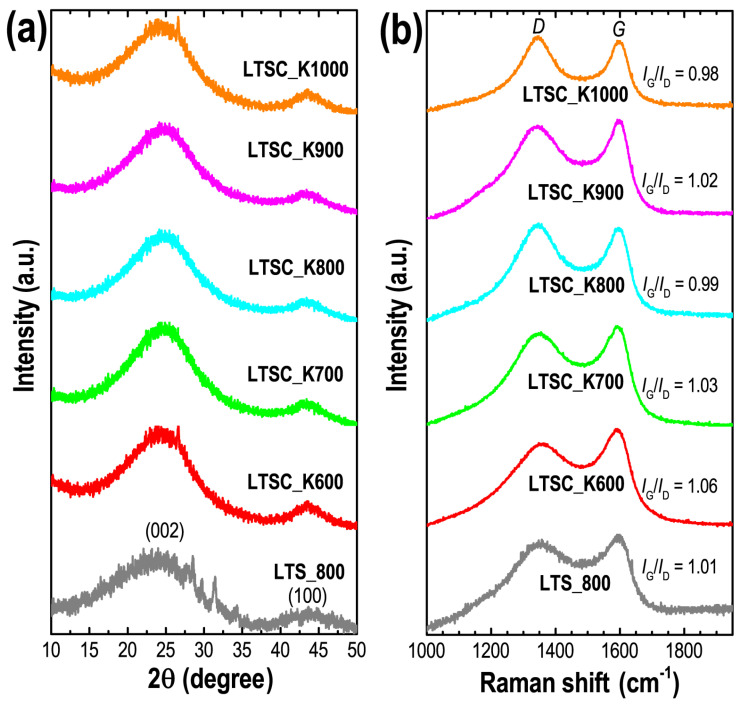
(**a**) pXRD and (**b**) Raman scattering spectra recorded at 25 °C for Lotus-seed–derived nanoporous carbon materials; LTS_800, LTSC_K600, LTSC_K700, LTSC_K800, LTSC_K900 and LTSC_K1000.

Using pXRD and Raman scattering spectroscopy, we studied the structure of the prepared carbon materials. All the samples show typical XRD patterns commonly observed in amorphous carbon materials (Figure 3a). Two broad diffraction peaks observed at diffraction angles of 24 and 43° can be attributed to the (002) and (100) plans of disordered graphite-like structures of amorphous carbon often realized in biomass-derived carbon materials [54]. Raman scattering spectra support the structural characteristics of these samples. Raman spectra contain two pronounced peaks located at ~1350 and 1595 cm^−1^, corresponding to the *D* (disorder or imperfect structure) and *G* (graphitic structure) bands of amorphous carbons (Figure 3b) [56]. The intensity ratios of *G* and *D* bands (*I_G_*/*I_D_*) are found in the range of 0.98 to 1.03, typically observed in amorphous carbon with partial graphitic structures. The presence of defects (in the form of micropore structures) in the carbon matrix is advantageous to increase the specific surface area and hence the capacitance performance. On the other hand, graphitic carbon structure contributes to enhancing the conductivity [57].

**Figure 4:**
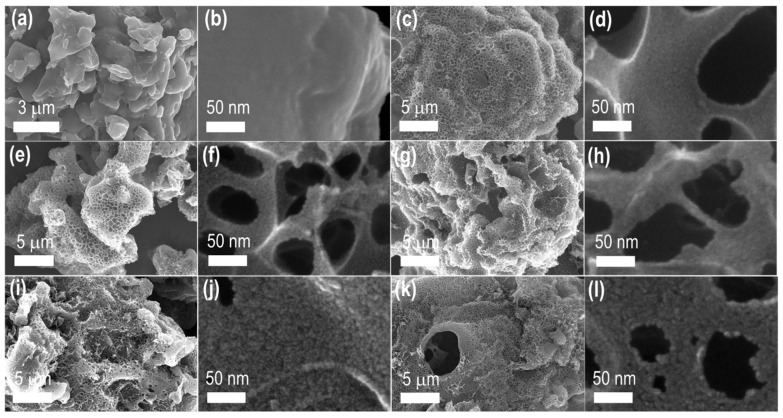
SEM observations of the Lotus-seed–derived carbon materials: (**a**,**b**) SEM images of LTS_800, (**c**,**d**) SEM images of LTSC_K600, (**e**,**f**) SEM images of LTSC_K700, (**g**,**h**) SEM images of LTSC_K800, (**i**,**j**) SEM images of LTSC_K900 and (**k**,**l**) SEM images of LTSC_K1000.

The surface morphology of the carbon materials was investigated by SEM observations. Figure 4 shows typical SEM images of the directly carbonized (LTS_800: Figure 4a,b) and KOH-activated samples (LTSC_K600, Figure 4c,d; LTSC_K700, Figure 4e,f; LTSC_K800, Figure 4g,h; LTSC_K900, Figure 4i,j; and LTSC_K1000, Figure 4k,l). Additional SEM images are supplied in Supplementary Materials Figures S2–S7. Micron-sized irregular-shaped particles (granules) are common in all the samples. Pores are not noticeable in the low- and high-resolution SEM images of the LTS_800 sample, demonstrating the lack of surface porosity. However, abundant large-size macropores with channel-like structures are observed in the KOH-activated samples (Figure 4c,e,g,i,k and Supplementary Materials Figures S2–S7). The frameworks of these macroporous channels comprised well-developed micro- and mesopore structures due to the KOH activation, suggesting the hierarchical pore structures. Therefore, the KOH-activated samples are expected to display a high specific surface area and well-defined pore size distribution with a large pore volume advantageous in high-energy-storage supercapacitors.

**Figure 5:**
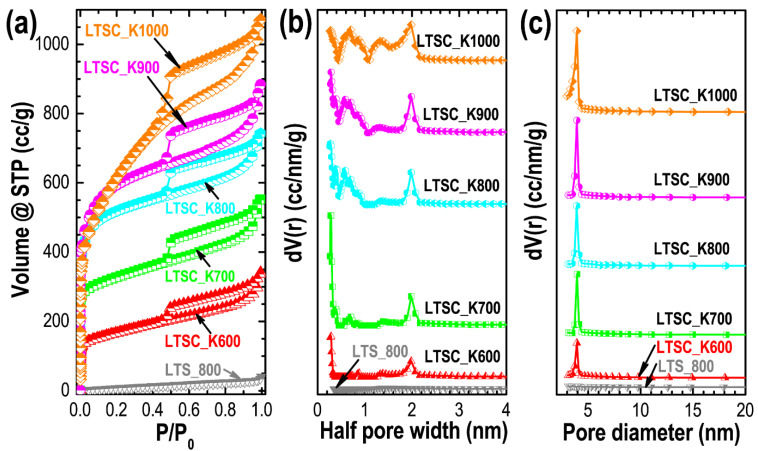
Textural properties by nitrogen sorption measurements. (**a**) Nitrogen adsorption/desorption isotherms of LTS_800, LTSC_K600, LTSC_K700, LTSC_K800, LTSC_K900 and LTSC_K1000 samples; (**b**) pore-size-distribution profiles from the density functional theory (DFT) method; and (**c**) pore-size-distribution profiles obtained from the Barrett–Joyner–Halenda (BJH) model.

Nitrogen sorption measurements were performed to study the textural properties (porosity) of the prepared carbon materials. The nitrogen uptake in the LTS_800 sample is low, and the isotherm shows Type-III sorption behavior corresponding to the nonporous materials, which supports the SEM observation that the directly carbonized sample lacks well-developed porosity. While all the KOH-activated samples show mixed Type-I/Type-IV adsorption isotherms (Figure 5a). Significant nitrogen adsorption at a low relative pressure (P/P0 < 0.1), followed by a gradual nitrogen uptake at high relative pressure with a clear hysteresis loop, demonstrates the presence of hierarchically bimodal micro- and mesopore architectures. Substantial nitrogen uptake in the low relative pressure region corresponds to micropore filling, while the hysteresis loop at high relative pressure is due to capillary condensation occurring in the mesopores [24,58,59]. Careful observation of the sorption isotherms reveals that the nitrogen uptake in the low relative pressure increases with the carbonization temperature up to 900 °C and then decreases. However, the size of the hysteresis loop monotonously increases with the temperature, suggesting that the micropore coalescence takes place at 1000 °C, leading to more mesoporous structure formation in the LTSC_K1000 sample. The pore-size-distribution profiles determined from the density functional theory (DFT) method (Figure 5b) and Barrett–Joyner–Halenda (BJH) model (Figure 5c) show prominent peaks in the micro- and mesopore region, thus further confirming the presence of hierarchically pore architectures in the KOH-activated samples. The textural properties summarized in Table 1 clearly show the crucial role of the carbonization temperature in porosity development. An increase in temperature monotonously increases the total specific surface area. However, microporosity reaches a maximum at 900 °C and then decreases, caused by the micropore coalescence, leading to the formation of mesopores, demonstrating that the LTSC_K900 sample has a much larger electrochemically accessible surface area and appropriate pore size distribution (majority micropores), enhancing the electrolyte ion adsorption and thus improving the energy storage capacity.

**Figure 6:**
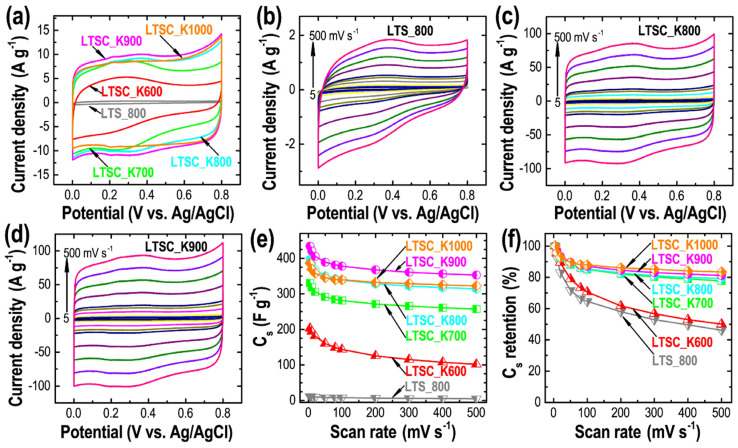
Cyclic voltammetry study of the Lotus-seed–derived carbon materials. (**a**) The CV profiles at a fixed scan rate of 50 mV s^−1^, and the CV profiles at different scan rates from 5 to 500 mV s^−1^ for (**b**) LTS_800, (**c**) LTSC_K800 and (**d**) LTSC_K900 as typical examples. (**e**) The calculated *C*_s_ vs. scan rate. (**f**) The capacitance retention.

Encouraged by the hierarchically porous architectures, ultrahigh surface area, well-developed porosity, large pore volume and self-nitrogen doping, we explored the Lotus-seed–derived activated carbons as the electrode materials for the electrical double-layer supercapacitor applications. The CV curves recorded at a fixed scan rate of 50 mV s^−1^ in a three-electrode system in aqueous 1M H_2_SO_4_ (Figure 6a) agree with the results obtained from the nitrogen sorption isotherms and are well correlated to the textural properties that increase microporosity increases the total integral current. The LTSC_K900 samples show the highest current output, demonstrating the highest energy-storage capacity among the studied samples. The rectangular shapes of the CV curves with weak humps in the range of 0.2–0.45 V, followed by a quick response to the current on the reversal of the potential sweep, suggest the dominance of the electrical double-layer capacitor charge storage mechanism characteristics of the carbon materials with some contribution of pseudocapacitance, due to the presence of the nitrogen and oxygen functionalities [6,23,49,60]. The CV profiles of the electrodes prepared from all the samples reveal that the integral current increases with the scan rate, while sustaining the semi-rectangular shape of the curves (Figure 6b–d and Supplementary Materials Figure S8). Note that the weak peaks have come from the pseudocapacitive behavior of the nitrogen and oxygen functionality decrease in the carbon samples prepared at higher temperatures due to loss of nitrogen- and oxygen content (Figure 2a and Figure 4b–d and Supplementary Materials Figure S8b–d). The *C*_s_ of the electrode materials calculated by using Equation (6) shows that the directly carbonized sample, due to the lack of electrochemically accessible micropores, possesses very low specific capacitance as compared to the KOH-activated samples. The optimal sample, LTSC_K900, achieved a high *C*_s_ of ca. 434.5 F g^−1^ at 5 mV s^−1^, which can be attributed to the outstanding surface area caused due to the presence of hierarchically micro- and mesopore architecture and self-nitrogen doping. Among the KOH-activated samples, the *C*_s_ follow the order LTSC_K900 > LTSC_K800 > LTSC_K1000 > LTSC_K700 > LTSC_K600, which is in good agreement with the microporosity of the materials (Table 1). Furthermore, the electrodes prepared from the KOH-activated samples show outstanding capacitance retentions at a high scan rate, 500 mV s^−1^ (Figure 6f). Interestingly, the *C*_s_ retention of LTSC_K1000 (83.4%) is better than it is for LTSC_K900 (81.2%), suggesting the fast electrolyte ion diffusion through the mesopore channels, even at the high potential sweep.

**Figure 7:**
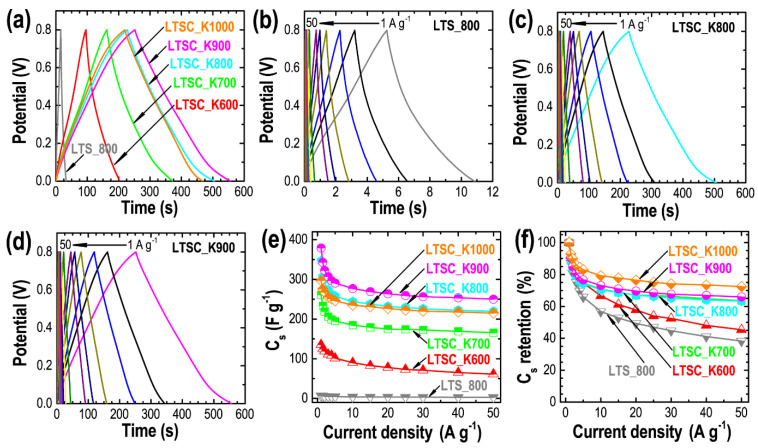
Results obtained from the GCD measurements. (**a**) The GCD curves recorded at a constant current density of 1 A g^−1^ for LTS_800, LTSC_K600, LTSC_K700, LTSC_K800, LTSC_K900 and LTSC_K1000, and GCD curves vs. current density for (**b**) LTS_800, (**c**) LTSC_K800 and (**d**) LTSC_K900. (**e**) The *C*_s_ vs. current density. (**f**) The corresponding rate performance.

**Figure 8:**
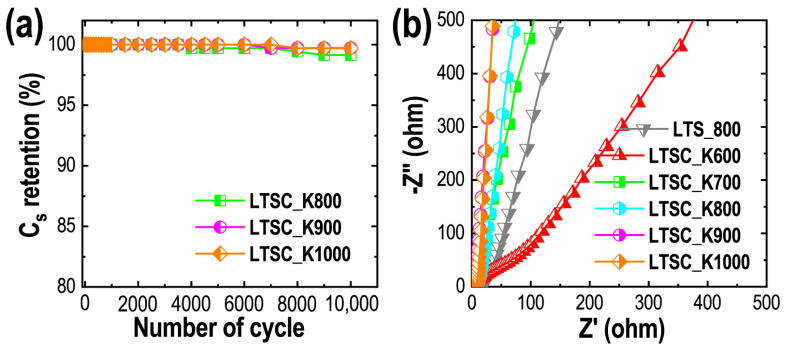
(**a**) Cyclic stability performance of LTSC_K800, LTSC_K900 and LTSC_K1000 electrodes at 50 A g^−1^. (**b**) Nyquist plots obtained from the EIS measurements for all the samples (LTS_800, LTSC_K600, LTSC_K700, LTSC_K800, LTSC_K900 and LTSC_K1000).

Figure 7 describes the results obtained from the GCD measurements between 0 and 0.8 V, at different current densities, from 1 to 50 A g^−1^. Figure 7a shows the GCD curves measured at a constant current density of 1 A g^−1^ for LTS_800 and the KOH-activated samples. The GCD curves recorded at a high current density of 50 A g^−1^ are shown in Supplementary Materials Figure S9a. The symmetrical quasi-triangular shaped GCD curves with the linear decay during discharging indicate the ideal capacitive behavior of the electrode materials with well-balanced charge storage [60,61]. The discharge time follows the order of LTSC_K900 > LTSC_K800 > LTSC_K1000 > LTSC_K700 > LTSC_K600 > LTS_800, which is correlated to the micropore surface area of the materials (Table 1). A small deviation from the triangular shape with a small voltage drop is caused due to nitrogen and oxygen functional groups. Note that the discharge time of the directly carbonized sample is far less than the discharge times of the KOH-activated samples, suggesting better energy-storage capacity of the activated samples. As expected from the porosity properties, the LTSC_K900 sample has an extended discharge time indicating the highest energy storage capacity among the other activated samples studied. The GCD curves recorded at different current densities (1 to 50 A g^−1^) show that the quasi-triangular shape of the curve is sustained even at a high current density of 50 A g^−1^ (Figure 7b–d and Supplementary Materials Figure S9b–d), indicating the fast ion transfer to the electrode surface for the formation of an electrical double layer with a good rate performance and well-balanced storage of the charges. Figure 8e shows the *C*_s_ calculated by using Equation (7). Due to the electrochemically accessible high microporous surface area, the LTSC_K900 sample shows the highest specific capacitance of ca. 379.2 F g^−1^, while the nonporous sample obtained by direct carbonization show only 7.1 F g^−1^ at 1 A g^−1^. The rate capability of the electrodes prepared from the carbon materials obtained at higher carbonization temperatures (700–1000 °C) shows better performance than the directly carbonized sample. The capacitance retentions were ca. 63.5% (LTSC_K700), 63.3% (LTSC_K800), 65.9% (LTSC_K900) and 72.4% (LTSC_K1000) at a high current density of 50 A g^−1^, thus suggesting the excellent rate performance essentially required for high-performance supercapacitors. Our materials’ overall electrochemical supercapacitance performance is better than the commercial activated carbons, for which *C*_s_ is reported in the range of ~100 F g^−1^ [52,62], and comparable to or better than the performance of nanoporous activated carbon materials derived from other biomass precursors, such as Washnut, Lapsi and Jackfruit seed; corncob; bamboo; and others (Supplementary Materials Table S1).

Cycling stability is a crucial parameter to evaluate the performance of supercapacitors. Here we have studied the cycle performance of the selected electrodes prepared by using LTSC_K800, LTSC_K900 and LTSC_K1000 samples at a fixed current density of 50 A g^−1^. As commonly observed in the electrical double-layer capacitive materials, all the electrodes display outstanding long-cycle performance sustaining more than 99% capacitance after the successive 10,000 charging/discharging cycles (Figure 8a). The outstanding cycle performance can be attributed to the hierarchically porous architecture with micro- and mesopore structures that contributes to the fast electrolyte ion diffusion to the electrode surface [63,64].

EIS measurements reveal the charge storage mechanism and electrolyte ions’ diffusion kinetics at the electrode surface. The straight vertical lines at the low-frequency region in the Nyquist plots (negative of imaginary part versus real part of the complex impedance) are the characteristic of typical carbon materials with double-layer capacitive behavior (Figure 8b) [65]. Depending on the sample, the electrode has a different ion diffusion resistance; for example, the LTSC_K600 sample shows non-vertical lines in the low frequency, due to the relatively high ion diffusion resistance. Due to the nitrogen and oxygen functionalities, the electrode prepared by using carbon samples carbonized at the lower temperature display weak semicircular response, suggesting a minimal charge-transfer resistance and hence a high efficiency of the ion diffusion. The values of the equivalent series’ resistance (ESR: often interpreted as the sum of the bulk electrolyte resistance, the electrode resistance and contact resistance of electrode and current collector) estimated from the intersection point of the imaginary and real part at high frequency are low, indicating good conductivity of the prepared carbon materials. They are ca. 5.12, 5.10, 4.85, 4.62 and 4.61 Ω for LTSC_K600, LTSC_K700, LTSC_K800, LTSC_K900 and LTSC_K1000, respectively. The EIS results demonstrate that the differences in the energy-storage capacity of the KOH-activated samples are caused mainly due to the different porosity properties (surface area and pore volumes).

## 4. Conclusions

In conclusion, we studied the performance of the self-nitrogen-doped nanoporous carbon materials prepared by the KOH activation of the biochar of the Lotus-seed powder by measuring the electrochemical supercapacitance in an aqueous electrolyte (1 M H_2_SO_4_) in a three-electrode system. Activated carbon materials with nanoporous bimodal pore structures comprising micro- and mesopores and self-nitrogen doping were prepared by the KOH activation of the biochar obtained by the pyrolysis of Lotus-seed powder at 300 °C. The Lotus-seed biochar was mixed with KOH and carbonized at different temperatures (600 to 1000 °C). Surface textural properties show the crucial role of the carbonization temperature on the porosity. An increase in temperature increases the total specific surface area. However, the microporosity reaches a maximum at 900 °C and declines due to the micropore coalescence at high temperatures. The surface area and pore volume were calculated in the range of 1059.6 to 2489.6 m^2^ g^−1^ and 0.819 to 2.384 cm^3^ g^−1^, respectively. The amorphous carbons materials with partial graphitic structure contained a maximum of 3.28 atom% nitrogen content and possessed hierarchically micro- and mesoporous structures. The electrode prepared from the optimal sample performed excellently as the electrical double-layer capacitive material. The electrode achieved a high specific capacitance of ca. 379.2 F g^−1^ at 1 A g^−1^ current density, excellent rate performance sustaining 65.9% capacitance retention at 50 A g^−1^ and extraordinary long cycle life with only 0.3% capacity loss after 10,000 subsequent charging/discharging cycles. Thus, the electrochemical results demonstrate that *Nelumbo nucifera* seed, an agro-waste, exemplifies a novel yet low-cost precursor for the large-scale production of hierarchically porous carbon materials with self-nitrogen doping, having significant potential as the electrical double-layer capacitor electrode material in the applications of high-performance energy-storage supercapacitors.

## Figures and Tables

**Table 1:** Textural properties of Lotus-seed–derived carbon materials carbonized at different temperatures.

Sample	*SSA* (m^2^ g^−1^)	*S*_micro_ (m^2^ g^−1^)	*S*_meso_ (m^2^ g^−1^)	*V*_p_ (cm^3^ g^−1^)	*V*_micro_ (cm^3^ g^−1^)	*W*_p_ (nm)	*D*_p_ (nm)
LTS_800	46.1	18.8	27.3	0.102	0.044	-	3.09
LTSC_K600	1059.6	824.5	235.1	0.819	0.472	0.285	3.92
LTSC_K700	1878.4	1556.1	322.3	1.232	0.775	0.286	3.93
LTSC_K800	2236.6	1891.3	345.3	1.499	1.034	0.274	3.91
LTSC_K900	2330.1	1905.7	424.4	1.793	1.206	0.286	3.92
LTSC_K1000	2489.3	1725.6	763.7	2.384	1.488	0.705	3.93

*SSA* = total specific surface area; *S*_micro_ = micropore surface area; *V*_p_ = total pore volume; *V*_micro_ = pore volume from micropores; *W*_p_ = average half pore width, as obtained from the DFT model; *D*_p_ = average pore diameter obtained from the BJH analysis.

## Data Availability

The data presented in this study are available on request from the corresponding author.

## Supplementary Materials

The following are available online at https://www.mdpi.com/article/10.3390/nano11123175/s1. Figure S1: (**a**) FTIR spectra, and (**b**) XPS O 1s spectra with the deconvoluted peaks of the directly carbonized sample, LTS_800, and KOH activated samples; LTSC_K600, LTSC_K700, LTSC_K800, LTSC_K900, and LTSC_K1000. Figure S2: Additional SEM images of LTS_800. Figure S3: Additional SEM images of LTSC_K600. Figure S4: Additional SEM images of LTSC_K700. Figure S5: Additional SEM images of LTSC_K800. Figure S6: Additional SEM images of LTSC_K900. Figure S7: Additional SEM images of LTSC_K1000. Figure S8: (**a**) The CV curves of all the samples at a fixed scan rate of 5 mV s^−1^ recorded at 25 °C, and the CV curves vs. scan rates for (**b**) LTSC_K600, (**c**) LTSC_K700, and (**d**) LTSC_K1000 systems. Figure S9: (**a**) The GCD curves at a constant current density of 50 A g^−1^, and the GCD curves vs. current density for (**b**) LTSC_K600, (**c**) LTSC_K700, and (**d**) LTSC_K1000. Table S1: Comparison of the electrochemical supercapacitance of the KOH activated *Nelumbo nucifera* (Lotus) seed carbon materials with activated carbon materials derived from other biomass.
