# Supplementary material for: Nelumbo nucifera Seed–Derived Nitrogen-Doped Hierarchically Porous Carbons as Electrode Materials for High-Performance Supercapacitors
Source: Nanomaterials (Basel). 2021 Nov 23;11(12):3175. doi: 10.3390/nano11123175 (PMC8707477; doi:10.3390/nano11123175)
Supplement: Supplementary file 1 [file nanomaterials-11-03175-s001.zip › nanomaterials-1467257-supplementary.pdf]

nanomaterials

MDPI

Supplementary Materials: Nelumbo nucifera Seed—Derived Nitrogen-Doped Hierarchically Porous Carbons as Electrode Materials for High-Performance Supercapacitors

Lok Kumar Shrestha [1,*], Rekha Goswami Shrestha [1,*], Rashma Chaudhary [2], Raja Ram Pradhananga [2], Birendra Man Tamrakar [3], Timila Shrestha [2], Subrata Maji [1], Ram Lal Shrestha [2,*] and Katsuhiko Ariga [1,4]

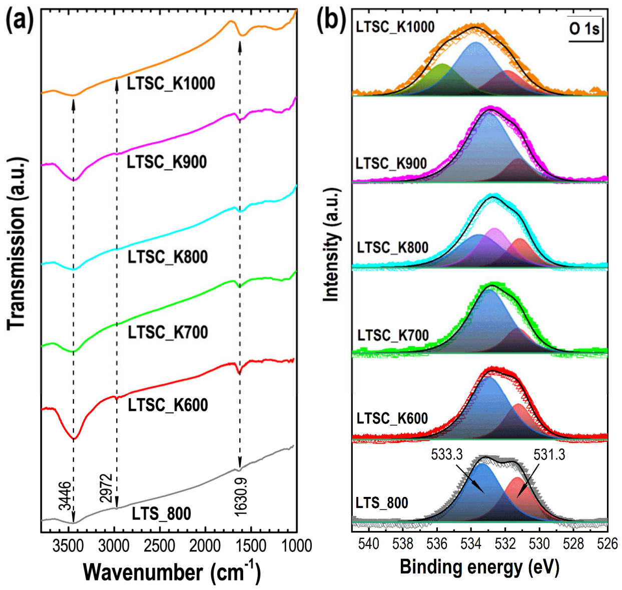

Figure S1. (**a**) FTIR spectra, and (**b**) XPS O 1s spectra with the deconvoluted peaks of the directly carbonized sample, LTS_800, and KOH activated samples; LTSC_K600, LTSC_K700, LTSC_K800, LTSC_K900, and LTSC_K1000.

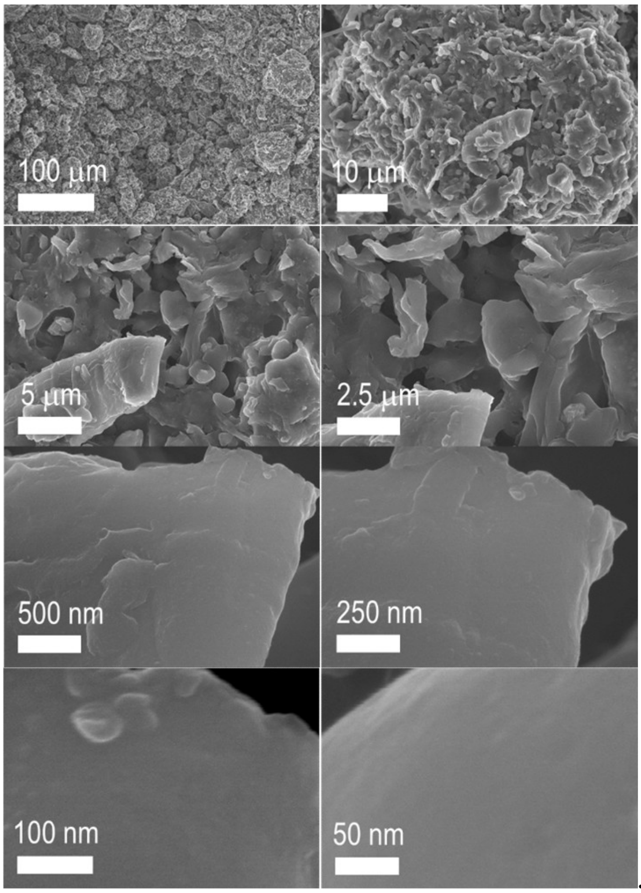

Figure S2. Additional SEM images of the LTS_800.

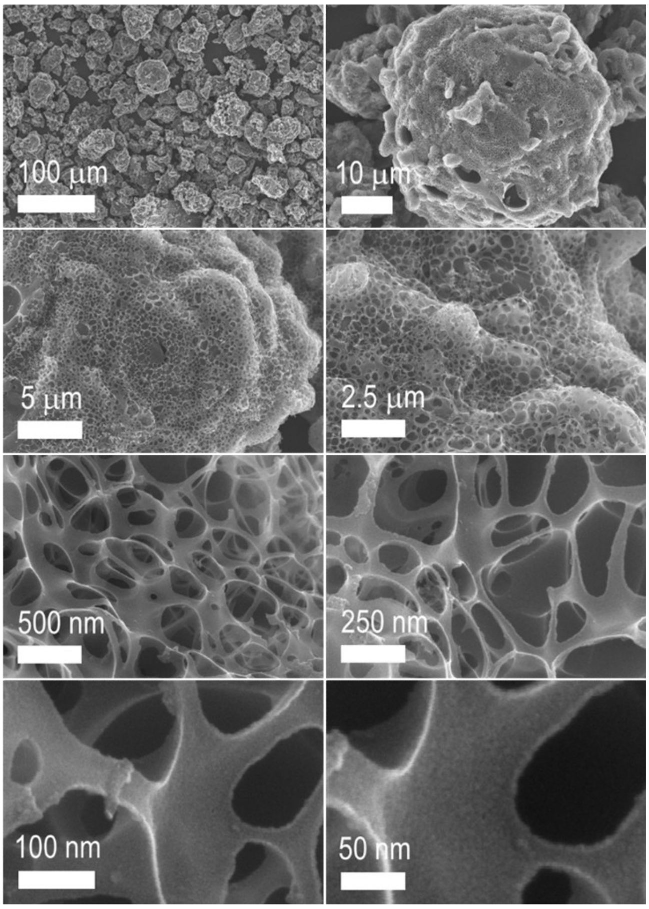

Figure S3. Additional SEM images of the LTSC_K600.

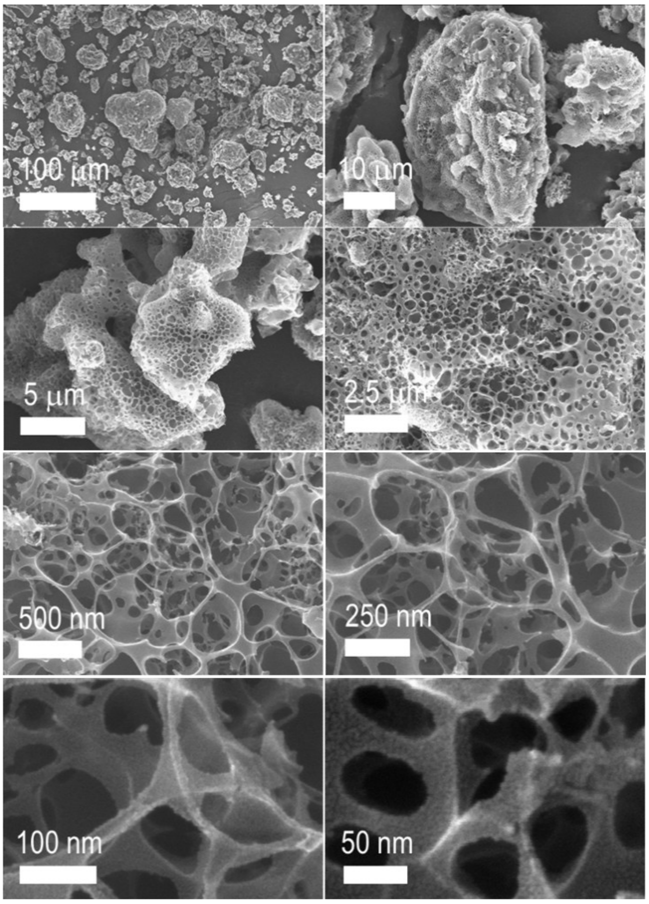

Figure S4. Additional SEM images of the LTSC_K700.

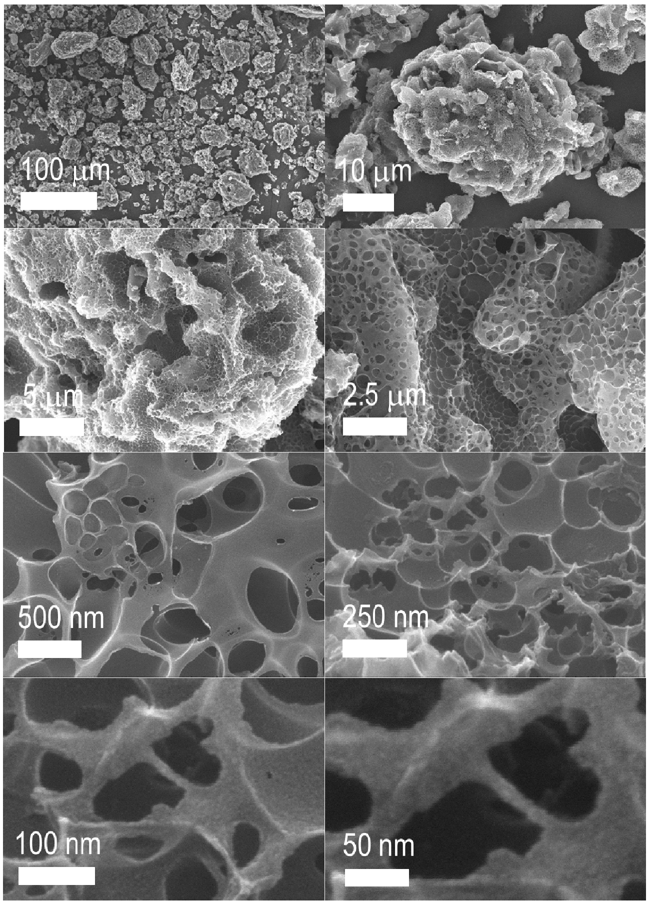

Figure S5. Additional SEM images of the LTSC_K800.

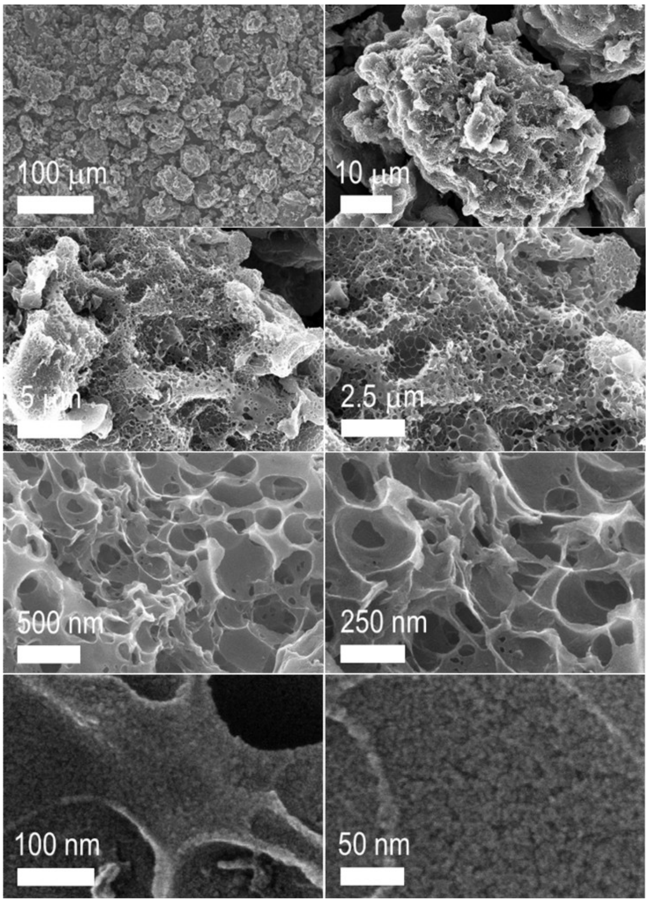

Figure S6. Additional SEM images of the LTSC_K900.

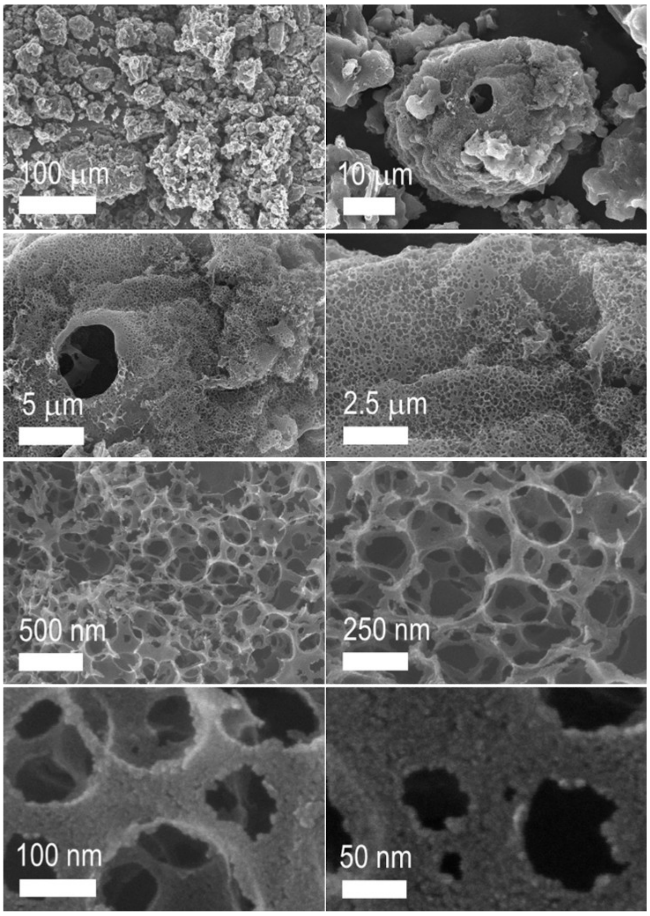

Figure S7. Additional SEM images of the LTSC_K1000.

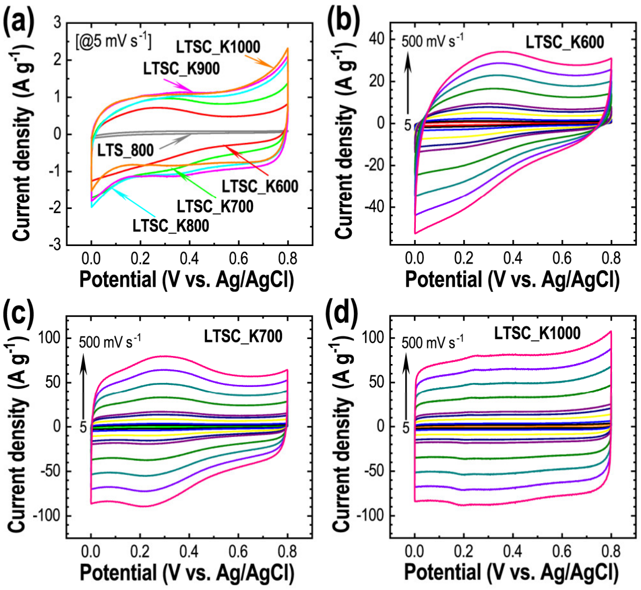

Figure S8. (**a**) The CV curves of all the samples at a fixed scan rate of 5 mV s^{-1} recorded at 25 °C, and the CV curves *vs.* scan rates for (**b**) LTSC_K600, (**c**) LTSC_K700, and (**d**) LTSC_K1000 systems.

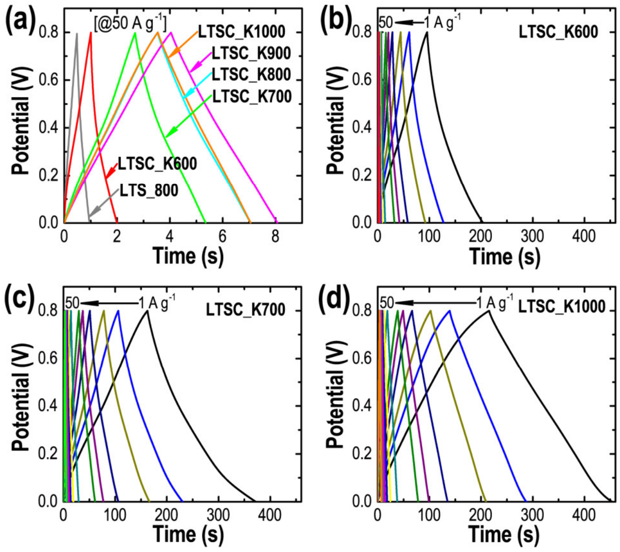

Figure S9. (**a**) The GCD curves at a constant current density of 50 A g^{-1}, and the GCD curves *vs*. current density for (**b**) LTSC_K600, (**c**) LTSC_K700, and (**d**) LTSC_K1000.

Table S1. Comparison of the electrochemical supercapacitance of the KOH activated *Nelumbo nucifera* (Lotus) seed carbon materials with activated carbon materials derived from other biomass.

Carbon precursors	T (°C)	Specific Surface Area (m^2 g^{-1})	N-content (at%)	Electrolyte	Current density (A g^{-1})	C_s (F g^{-1})	Potential window (V)	Cycle stability (%)	Reference
Lotus seed (KOH activated)	900	2330.1	0.91	1 M H_2SO_4	1	379.2	0.8	99% (10000 cycles)	This work
Albizia flowers	900	2757.63	1.34	6 M KOH	0.5	406	1.0	94% (10000 cycles)	[1]
Jackfruit seed (KOH activated)	900	2104.3	---	1 M H_2SO_4	1	323.8	0.8	97% (10000 cycles)	[2]
Jackfruit seed ($ZnCl_2$ activated)	800	1340.4	---	1 M H_2SO_4	1	261.3	0.8	99.4% (10000 cycles)	[3]
Washnut seed (KOH activated)	900	2034.9	---	1 M H_2SO_4	1	288.7	0.8	98% (10000 cycles)	[4]
Washnut seed ($ZnCl_2$ activated)	800	1309	---	1 M H_2SO_4	1	225.1	0.8	98% (10000 cycles)	[5]
Lotus seed ($ZnCl_2$ activated)	800	1316.7	---	1 M H_2SO_4	1	272.9	0.8	99.2% (10000 cycles)	[6]
Lapsi seed	700	2272	---	1 M H_2SO_4	1	284.0	0.8	99% (10000 cycles)	[7]
Corncob	400	1288	---	1 M H_2SO_4	1	261.6	0.8	96% (10000 cycles)	[8]
Cotton seed husk	800	584.49	---	6 M KOH	0.5	238	1.0	95.4% (6000 cycles)	[9]
Cottonseed hull	800	2573	8.44	6 M KOH	0.5	340	1.0	91% (5000 cycles)	[10]
Bio-decomposited product	800	3142	---	6 M KOH	0.05	209	1.0	97% (10000 cycles)	[11]
Bamboo (H_3PO_4 activated)	400	1431	---	1 M H_2SO_4	1	206.0	0.8	92.6% (1000 cycles)	[12]
Lignocellulose carbon	700	341	---	1 M NaCl	1	172.9	1.0	---	[13]
Biomass-derived lignin	800	559	---	6 M KOH	0.5	348	1.0	96%(10000 cycles)	[14]
Kraft lignin	800	1204	---	6 M KOH	0.1	155	1.2	94% (6000 cycles)	[15]
salvia splendens	800	1051	2.52	6 M KOH	1	294	1.0	94.7% (20000 cycles)	[16]
Quinoa	800	2597	1.97	6 M KOH	1	330	1.0	93% (10000 cycles)	[17]
Wood sawdust	850	2294	---	6 M KOH	0.5	225	1.0	94.2% (10000 cycles)	[18]
Wood	---	1123.9	---	1 M H_2SO_4	0.5	260	1.0	98% (5000 cycles)	[19]
Metaplexis Japonica seed	850	2041.8	2.5	6 M KOH	1	401.5	1.0	99.7% (20000 cycles)	[20]
Metaplexis Japonica shell	800	956.3	8	6 M KOH	2	457	1.6	97% (20000 cycles)	[21]
Commercial AC	---	879.8	---	2 M NaOH	1	427	1.0	---	[22]

(Calgon Carbon, USA)								
Commercial AC (YP-50)	---	1724	---	20 mol Kg^{-1} LiTFSI	1	118	2.4	61% (10000 cycles)	[23]

Reference

1. Wu, F.; Gao, J.; Zhai, X.; Xie, M.; Sun, Y.; Kang, H.; Tian, Q.; Qiu, H. Hierarchical Porous Caron Microrods Derived from Albizia Flower for High Performance Supercapacitors. *Carbon* **2019**, *147*, 242–251.
2. Maji, S.; Chaudhary, R.; Shrestha, R.G.; Shrestha, R.L.; Demir, B.; Searles, D.J.; Hill, J.P.; Yamauchi, Y.; Ariga, K.; Shrestha, L.K. High-Performance Supercapacitor Materials Based on Hierarchically Porous Carbons Derived from *Artocarpus heterophyllus* Seed. *ACS Appl. Energy Mater.* **2021**, (In press: https://doi.org/10.1021/acsaem.1c02051)
3. Chaudhary, R.; Maji, S.; Shrestha, R. G.; Shrestha, R. L.; Shrestha, T.; Ariga, K.; Shrestha, L. K. Jackfruit Seed-Derived Nanoporous Carbons as the Electrode Material for Supercapacitors. *C J. Carbon Res.* **2020**, *6*, 73.
4. Shrestha, R. L.; Chaudhary, R.; Shrestha, R. G.; Shrestha, T.; Maji, S.; Ariga, K.; Shrestha, L. K. Washnut Seed-Derived Ultrahigh Surface Area Nanoporous Carbons as High Rate Performance Electrode Material for Supercapacitors. *Bull. Chem. Soc. Jpn.* **2021**, *94*, 565–572.
5. Shrestha, R. L.; Shrestha, T.; Tamrakar, B. M.; Shrestha, R. G.; Maji, S.; Ariga, K.; Shrestha, L. K. Nanoporous Carbon Materials Derived from Washnut Seed with Enhanced Supercapacitance. *Materials* **2020**, *13*, 2371.
6. Shrestha, R. L.; Chaudhary, R.; Shrestha, T.; Tamrakar, B. M.; Shrestha, R. G.; Maji, S.; Hill, J. P.; Ariga, K.; Shrestha, L. K. Nanoarchitectonics of Lotus Seed Derived Nanoporous Carbon Materials for Supercapacitor Applications. *Materials* **2020**, *13*, 5434.
7. Shrestha, L. K.; Shrestha, R. G.; Maji, S.; Pokharel, B. P.; Rajbhandari, R.; Shrestha, R. L.; Pradhananga, R. R.; Hill, J. P.; Ariga, K. High Surface Area Nanoporous Graphitic Carbon Materials Derived from Lapsi Seed with Enhanced Supercapacitance. *Nanomaterials* **2020**, *10*, 728.
8. Adhikari, M. P.; Adhikari, R.; Shrestha, R. G.; Rajendran, R.; Adhikari, L.; Bairi, P.; Pradhananga, R. R.; Shrestha, L. K.; Ariga, K. Nanoporous Activated Carbons Derived from Agro-Waste Corncob for Enhanced Electrochemical and Sensing Performance. *Bull. Chem. Soc. Jpn.* **2015**, *88*, 1108–1115.
9. Liu, Y.; Shi, Z.; Gao, Y.; An, W.; Cao, Z.; Liu, J. Biomass-Swelling Assisted Synthesis of Hierarchical Porous Carbon Fibers for Supercapacitor Electrodes. *ACS Appl. Mater. Interfaces* **2016**, *8*, 28283–28290.
10. Jiang, Y.; Zhang, Z.; Zhang, Y.; Zhou, X.; Wang, L.; Yasin, A.; Zhang, L. Bioresource derived porous carbon from cottonseed hull for removal of triclosan and electrochemical application. *RSC Adv.* **2018**, *8*, 42405–42414.
11. Zhu, Y.; Chen, M.; Zhang, Y.; Zhao, W.; Wang, C. A Biomass-Derived Nitrogen-Doped Porous Carbon for High-Energy Supercapacitor. *Carbon* **2018**, *140*, 404–412.
12. Shrestha, L.K.; Adhikari, L.; Shrestha, R.G.; Adhikari, M.P.; Adhikari, R.; Hill, J.P.; Pradhananga, R.R.; Ariga, K. Nanoporous carbon materials with enhanced supercapacitance performance and non-aromatic chemical sensing with C1/C2 alcohol discrimination. *Sci. Technol. Adv. Mater.* **2016**, 17, 483–492.
13. Lu, T.; Xu, X.; Zhang, S.; Pan, L.; Wang, Y.; Alshehri, S. M.; Ahamad, T.; Kim, M.; Na, J.; Hossain, Md. S. A.; Shapter, J. G.; Yamauchi, Y. High-Performance Capacitive Deionization by Lignocellulose-Derived Eco-Friendly Porous Carbon Materials. *Bull. Chem. Soc. Jpn.* **2020**, *93*, 1014–1019.
14. Cao, M.; Wang, Q.; Cheng, W.; Huan, S.; Hu, Y.; Niu, Z.; Han, G.; Cheng, H.; Wang, G. A Novel Strategy Combining Electrospraying and One-Step Carbonization for the Preparation of Ultralight Honeycomb-like Multilayered Carbon from Biomass-Derived Lignin. *Carbon* **2021**, *179*, 68–79.
15. Schlee, P.; Hosseinaei, O.; Baker, D.; Ladmér, A.; Tomani, P.; Mostazo-López, M. J.; Cazorla-Amorós, D.; Herou, S.; Titirici, M.-M. From Waste to Wealth: From Kraft Lignin to Free-standing Supercapacitors. *Carbon* **2019**, *145*, 470–480.
16. Liu, B.; Yang, M.; Chen, H.; Liu, Y.; Yang, D.; Li, H. Graphene-Like Porous Carbon Nanosheets Derived from *salvia splendens* for High-Rate Performance Supercapacitors. *J. Power Sources* **2018**, *397*, 1–10.
17. Sun, Y.; Xue, J.; Dong, S.; Zhang, Y.; An, Y.; Ding, B.; Zhang, T.; Dou, H.; Zhang, X. Biomass-derived porous carbon electrodes for high-performance supercapacitors. *J. Mater. Sci.* **2020**, *55*, 5166–5176.
18. Huang, Y.; Peng, L.; Liu, Y.; Zhao, G.; Chen, J. Y.; Yu. G. Biobased Nano Porous Active Carbon Fibers for High-Performance Supercapacitors. *ACS Appl. Mater. Interface* **2016**, *8*, 15205–15215.
19. Chen, Z.; Zhou, H.; Hu, Y.; Lai, H.; Liu, L.; Zhong, L.; Peng, X. Wood-Derived Lightweight and Elastic Carbon Aerogel for Pressure Sensing and Energy Storage. *Adv. Funct. Mater.* **2020**, *30*, 1910292.
20. Zhao, Y.; Dong, C.; Sheng, L.; Xiao, Z.; Jiang, L.; Li, X.; Jiang, M.; Shi, J. Heteroatom-Doped Pillared Porous Carbon Architectures with Ultrafast Electron and Ion Transport Capabilities under High Mass Loadings for High-Rate Supercapacitors. *ACS Sustainable Chem. Eng.* **2020**, *8*, 8664–8674.
21. Sheng, L.-Z.; Zhao, Y.-Y.; Hou, B.-Q.; Xiao, Z.-P.; Jiang, L.L.; Fan, Z.-J. N-Doped Layered Porous Carbon Electrodes with High Mass Loadings for High-Performance Supercapacitors. *New Carbon Materials* **2021**, *36*, 179–188.

22. Wickramaarachchi, W.A.M.K.P.; Minakshi, M.; Gao, X.; Dabare R.; Wong, K.W. Hierarchical Porous Carbon from Mango Seed Husk for Electro-Chemical Energy Storage. *Chem. Eng. J. Adv.* **2021**, *8*, 100158.
23. Liu, X.; Mi, R.; Yuan, L.; Yang, F.; Fu, Z.; Wang, C.; Tang, Y. Nitrogen-Doped Multi-Scale Porous Carbo for High Voltage Aqueous Supercapacitors. *Fornt. Chem.* **2018**, *6*, 475.
